# A Concomitant Cancer Diagnosis Is Associated With Poor Cardiovascular Outcomes Among Acute Myocardial Infarction Patients

**DOI:** 10.3389/fcvm.2022.758324

**Published:** 2022-02-17

**Authors:** Xiang Peng, Zhuozhong Wang, Muhua Cao, Yuqi Zheng, Ya'nan Tian, Li Yu, Wenjun Ni, Shanjie Wang, Zhifeng Qin, Suhong Zhao, Jinwei Tian, Bo Yu

**Affiliations:** ^1^Department of Cardiology, The Second Affiliated Hospital of Harbin Medical University, Harbin, China; ^2^The Key Laboratory of Myocardial Ischemia, Harbin Medical University, Ministry of Education, Harbin, China

**Keywords:** cancer, acute myocardial infarction, cardiovascular outcomes, percutaneous coronary intervention, conservative therapies

## Abstract

**Background and Aims:**

With the increasing coexistence of cardiovascular disease and cancer in contemporary clinical practice, studies on the outcomes in acute myocardial infarction (AMI) patients with cancer has not been systematically investigated. This study sought to investigated the effect of coexisting cancer on the treatment and clinical outcomes among AMI patients.

**Methods:**

We retrospectively integrated and analyzed cardiovascular data of 6,607 AMI patients between June 2016 and December 2019. Patients with cancer were compared with pair-matched cancer-naive patients. Cox proportional hazards models were constructed to compare the differences in outcomes.

**Results:**

Of 6,607 patients, 2.3% (*n* = 150) had been diagnosed with cancer. Patients with cancer were older (70.3 ± 10.0 vs. 63.9 ± 11.5 years, *P* < 0.001) and had a higher burden of comorbidities. Moreover, patients with cancer tended to receive clopidogrel (52.0 vs. 40.0%, *P* = 0.004) rather than ticagrelor (45.6 vs. 58.2%, *P* = 0.003) than those without cancer. After pairwise matching, patients with cancer were less likely to undergo in-hospital percutaneous coronary intervention (61.3 vs. 70.0%, *P* = 0.055). And after 3-year follow-up, the cumulative incidence of cardiovascular death (14.0 vs. 8.3%; adjusted HR, 1.93; 95% CI, 1.11–3.39; *P* = 0.021) among patients with cancer was significantly higher than that among the matched controls, a similar pattern was observed for the composite outcome of cardiovascular death, non-fatal myocardial infarction, and non-fatal stroke (16.0 vs. 10.3%; adjusted HR, 1.98; 95% CI, 1.21–3.26; *P* = 0.007). Moreover, patients with a historical cancer diagnosis within 5 years had a higher risk of cardiovascular ischemic events.

**Conclusions:**

AMI patients with a concomitant diagnosis of cancer tended to be treated with conservative therapies and were at substantially higher risk for adverse cardiovascular outcomes.

## Introduction

Cancer and cardiovascular disease are the leading causes of disease-related death worldwide, together accounting for nearly 70% (1). Due to earlier detection and modern treatment regimens, cancer-related mortality has decreased significantly (2), and two-thirds of patients with cancer can survive at least 5 years with the disease (3). Likewise, there has been a global decline in deaths from acute myocardial infarction (AMI) (4). Although cancer and cardiovascular disease are regarded as two distinct disease processes, there is a considerable overlap of risk factors for these diseases, such as advanced age, diabetes (5), smoking (6), and obesity (7). As life expectancy increases, non-cancer-related mortality from cardiovascular disease has become more important during cancer survivorship (8, 9), and cardiovascular disease has been shown to be the leading cause of death in cancer patients (10, 11).

When cancer patients present with AMI, their management poses unique challenges for clinicians. Many old and new emerging anti-cancer agents are associated with cardiovascular toxicities (12, 13). The lasting cardiovascular side effects of cancer treatments means that the compensatory reserve for acute clinical events such as AMI may also be reduced (14). At a cumulative (i.e., lifetime) dose of 400–450 mg/m^2^ doxorubicin, a 10% rate of heart failure can be expected among patients aged over 65 years (15). In addition, cancer is commonly associated with hematologic and coagulation abnormalities (16), which poses a major obstacle to percutaneous coronary intervention (PCI) and the use of antithrombotic agents. Unfortunately, patients with cancer are commonly excluded from randomized controlled trials exploring best practices for the treatment of AMI, leading to a scarcity of reliable data on clinical outcomes in this cohort to guide clinical decision-making, which compounds the dilemma faced by clinicians.

Therefore, in this retrospective cohort study, we analyzed the clinical characteristics, treatment patterns, and outcomes in AMI patients with cancer and sought to define the influence of cancer duration and treatment pattern on the cardiovascular outcomes.

## Materials and Methods

### Study Design and Patient Population

A retrospective, single-center study was performed at the Second Affiliated Hospital of Harbin Medical University, which was approved by the ethics committee of Harbin Medical University. The study procedures were conducted in compliance with the principles of the Declaration of Helsinki, and patient information was collected anonymously. All AMI patients from June 2016 to December 2019 were included in the study. Myocardial infarction (MI) was defined according to the fourth universal definition of MI (17). The population included in the final analysis consisted of 6,607 AMI patients. All detailed clinical data of those patients were collected from electronic medical records, including age, sex, type of malignancy, cardiovascular risk factors [smoking status, hypertension, hyperlipidemia, diabetes mellitus, and previous coronary heart disease (CHD)], treatment, and outcomes. During a 3-year follow-up period, patients were surveyed semi-annually *via* telephone about major adverse events using a standardized questionnaire.

### Outcomes

Our primary outcome was defined as cardiovascular mortality during follow-up. Secondary outcomes included all-cause mortality, major adverse cardiovascular and cerebrovascular events (MACCE), non-fatal MI, non-fatal stroke, and revascularization. MACCE is composed of cardiovascular death, non-fatal myocardial infarction, or non-fatal stroke.

### Statistical Analysis

For all statistical tests, a two-tailed *P*-value < 0.05 indicated statistical significance, and data analyses were performed using R version 3.6.2 software (R Institute Inc.). Continuous variables are presented as the means ± standard deviations (SDs) if normally distributed or presented as medians with interquartile ranges (IQRs) if non-normally distributed. discrete variables are presented as frequencies (percentages), and missing data were excluded from the summary statistic calculations. To evaluate the differences in baseline characteristics between unmatched groups, Student's *t-*test was used for nearly normally distributed continuous variables, the Wilcoxon rank-sum test was used for non-normally distributed continuous and ordinal discrete variables, and Categorical data have been compared using the χ^2^ or Fisher's exact test. Furthermore, to make the two groups comparable with regard to the vast majority of baseline characteristics, pairwise matching was performed *via* a greedy matching algorithm to match each pair of reference patients and patients with cancer according to the following restrictions: (1) age within 1 year, (2) sex, (3) hyperlipidemia status, (4) smoking status, and (5) diabetes status. The control group allowed a variable number of reference matches and a maximum of 4 matches per patient with cancer. Except for unpaired patients, each patient pair was used once in the further analyses. Comparisons between reference patients and patients with cancer were tested *via* the same test for baseline characteristics and outcomes. To evaluate the incremental relative risk increase among subgroups in the heterogeneity analysis, models were fit with an indicator for any history of cancer and with another indicator for the subgroup. Forest plots were drawn to analyze the heterogeneity of the effect of coexisting cancer on the event risk between subgroups.

## Results

### Patient Characteristics

A total of 6,607 AMI patients were included between June 2016 and December 2019. Among those patients, 150 (2.3%) had been diagnosed with cancer. According to the order of frequencies, the most prevalent malignancies were lung (31, 20.7%), colorectum (21, 14.0%), stomach (19, 12.7%), and breast (15, 10.0%) cancers (Supplementary Table S1).

The characteristics of the overall cohort and matched cohort are summarized in Table 1. Before matching, the group of patients with cancer was older (70.3 ± 10.0 vs. 63.9 ± 11.5 years, *P* < 0.001) and had higher proportions of patients with hyperlipidemia (31.3 vs. 22.7%, *P* = 0.018) and diabetes (38.3 vs. 24.2%, *P* < 0.001). The group of patients with cancer had a lower proportion of current smokers (31.1 vs. 48.7%, *P* < 0.001) but higher proportions of patients with comorbidities. Furthermore, the group of patients with cancer had higher proportions of patients with previous CHD (44.9 vs. 25.7%, *P* < 0.001), previous MI (21.8 vs. 11.0%, *P* < 0.001) and previous PCI (16.3 vs. 7.3%, *P* < 0.001) than the group without cancer. During hospitalization, patients with cancer tended to receive clopidogrel (52.0 vs. 40.0%, *P* = 0.004) rather than ticagrelor (45.6 vs. 58.2%, *P* = 0.003) given an aspirin background. In addition, matching was possible for 542 pairs of reference patients and patients with cancer, and those patients constituted our matched study groups. After controlling for these heterogeneous covariates, such as age, sex, diabetes, smoking habits, and hyperlipidemia, the baseline characteristics were similar between the groups after matching with the exception of higher proportions of patients with previous CHD (44.9 vs. 26.8%, *P* < 0.001), previous MI (21.8 vs. 11.1%, *P* = 0.001) and previous PCI (16.3 vs. 5.9%, *P* < 0.001) in the group of patients with cancer than in the matched controls. Moreover, patients with cancer were less likely to undergo in-hospital PCI (61.3 vs. 70.0%, *P* = 0.055).

**Table 1:** Clinical characteristics.

	**Unmatched**			**Matched**		
	**No cancer**	**Cancer**	***P-*value**	**No cancer**	**Cancer**	***P-*value**
***N***	6,457	150		542	150	
Age	63.9 ± 11.5	70.3 ± 10.0	<0.001	70.3 ± 9.4	70.3 ± 10.0	>0.999
Male	4,447 (68.9)	93 (62.0)	0.088	340 (62.7)	93 (62.0)	0.946
STEMI	4,156 (65.5)	85 (64.4)	0.861	331 (62.0)	85 (64.4)	0.681
BMI^a^	24.9 ± 3.7	24.5 ± 3.8	0.179	24.9 ± 3.6	24.5 ± 3.8	0.295
**Risk factors**						
Hypertension^a^	3,428 (53.2)	89 (60.1)	0.110	312 (57.7)	89 (60.1)	0.657
Hyperlipidemia^a^	1,462 (22.7)	46 (31.3)	0.018	160 (29.5)	46 (31.3)	0.753
Diabetes^a^	1,559 (24.2)	57 (38.3)	<0.001	194 (35.8)	57 (38.3)	0.648
Current smoker^a^	3,140 (48.7)	46 (31.1)	<0.001	170 (31.4)	46 (31.1)	>0.999
**Comorbidities**						
Coronary heart disease^a^	1,656 (25.7)	66 (44.9)	<0.001	145 (26.8)	66 (44.9)	<0.001
History of MI^a^	711 (11.0)	32 (21.8)	<0.001	60 (11.1)	32 (21.8)	0.001
History of stroke^a^	1,320 (20.5)	30 (20.4)	>0.999	142 (26.2)	30 (20.4)	0.183
History of PCI^a^	470 (7.3)	24 (16.3)	<0.001	32 (5.9)	24 (16.3)	<0.001
History of CABG^a^	18 (0.3)	1 (0.7)	0.349^b^	0 (0.0)	1 (0.7)	0.213^b^
Peripheral vascular disease^a^	158 (2.5)	10 (6.8)	0.002	19 (3.5)	10 (6.8)	0.127
Liver disease^a^	130 (2.0)	6 (4.1)	0.147	13 (2.4)	6 (4.1)	0.412
Chronic kidney disease^a^	239 (3.7)	14 (9.5)	<0.001	26 (4.8)	14 (9.5)	0.048
**Clinical presentation**						
LDL-C, umol/mL^a^	2.0 ± 5.6	1.8 ± 0.6	0.646	1.9 ± 0.8	1.8 ± 0.6	0.701
Troponin I, ng/mL^a^	2.1 (0.3–10.9)	1.7 (0.4–8.6)	0.687	2.1 (0.4–11.9)	1.7 (0.4–8.6)	0.456
Pro-BNP, pg/mL^a^	294.0 (82.0–1,156.0)	428.5 (102.8–428.5)	0.353	473.0 (119.0–2,018.0)	428.5 (102.8–428.5)	0.085
LVEF ≤ 40%	409 (6.3)	4 (2.7)	0.096	39 (7.3)	4 (2.7)	0.062
Diastolic cardiac dysfunction^a^	3,808 (63.2)	88 (62.9)	>0.999	356 (70.2)	88 (62.9)	0.119
**Angiographic presentation**						
Lesion location						
LM	313 (4.9)	8 (5.3)	0.935	41 (7.6)	8 (5.3)	0.430
LAD	3,979 (61.6)	89 (59.3)	0.628	341 (63.5)	89 (59.3)	0.403
LCX	1,729 (26.8)	31 (20.7)	0.114	142 (26.4)	31 (20.7)	0.182
RCA	3,266 (50.6)	70 (46.7)	0.387	286 (53.3)	70 (46.7)	0.182
TIMI flow 0 or 1 in any lesion	2,396 (37.1)	53 (35.3)	0.720	205 (38.2)	53 (35.3)	0.589
**In-hospital procedures**						
PCI	4,261 (66.0)	92 (61.3)	0.270	376 (70.0)	92 (61.3)	0.055
PTCA	849 (13.2)	17 (11.3)	0.597	47 (8.75)	17 (11.3)	0.422
Thrombus suction pipe	1,456 (22.6)	32 (21.3)	0.800	102 (19.0)	32 (21.3)	0.601
Thrombolysis^a^	268 (4.2)	6 (4.1)	>0.999	14 (2.6)	6 (4.1)	0.495
Aspirin^a^	6,285 (97.4)	143 (96.0)	0.439	523 (97.4)	143 (96.0)	0.525
Clopidogrel^a^	2,582 (40.0)	77 (52.0)	0.004	246 (45.8)	77 (52.0)	0.212
Ticagrelor^a^	3,760 (58.2)	68 (45.6)	0.003	285 (53.1)	68 (45.6)	0.130
Statin^a^	6,263 (97.0)	143 (96.0)	0.616	520 (96.8)	143 (96.0)	0.795
ACEI^a^	3,026 (46.9)	67 (45.3)	0.761	251 (46.7)	67 (45.3)	0.822
ARB^a^	174 (2.7)	6 (4.1)	0.455	19 (3.5)	6 (4.1)	0.961
Beta-blocker^a^	3,947 (61.1)	88 (59.5)	0.743	332 (61.8)	88 (59.5)	0.669
**In-hospital complications**						
Reinfarction^a^	5 (0.1)	0 (0.0)	>0.999	0 (0.0)	0 (0.0)	1
Malignant arrhythmia	204 (3.2)	2 (1.3)	0.301	25 (4.7)	2 (1.3)	0.107
Cardiogenic shock^a^	142 (2.2)	2 (1.3)	0.663	13 (2.4)	2 (1.3)	0.624
Cardiopulmonary arrest^a^	93 (1.4)	1 (0.7)	0.658	9 (1.7)	1 (0.7)	0.598
Death	154 (2.4)	2 (1.3)	0.571	15 (2.8)	2 (1.3)	0.471

*Values are mean ± SD, median (interquartile range) or n (%)*.

a *Include some missing values since some patients did not accept these examinations*.

b *Result of fisher's exact test*.

*ACEI, angiotensin-converting enzyme inhibitor; ARB, angiotensin receptor blocker; BMI, body mass index; CABG, coronary artery bypass grafting; LAD, left anterior descending artery; LDL-C, low-density lipoprotein cholesterol; LM, left main coronary artery; LVEF, left ventricular ejection fraction; MI, myocardial infarction; NT-proBNP, N-terminal pro–brain natriuretic peptide; PCI, percutaneous coronary intervention; PTCA, percutaneous transluminal coronary angioplasty; SD, standard deviation; STEMI, ST segment elevation myocardial infarction; TIMI, Thrombolysis In Myocardial Infarction*.

### Outcomes in the Cancer and Matched Non-cancer Groups

**Figure 1:**
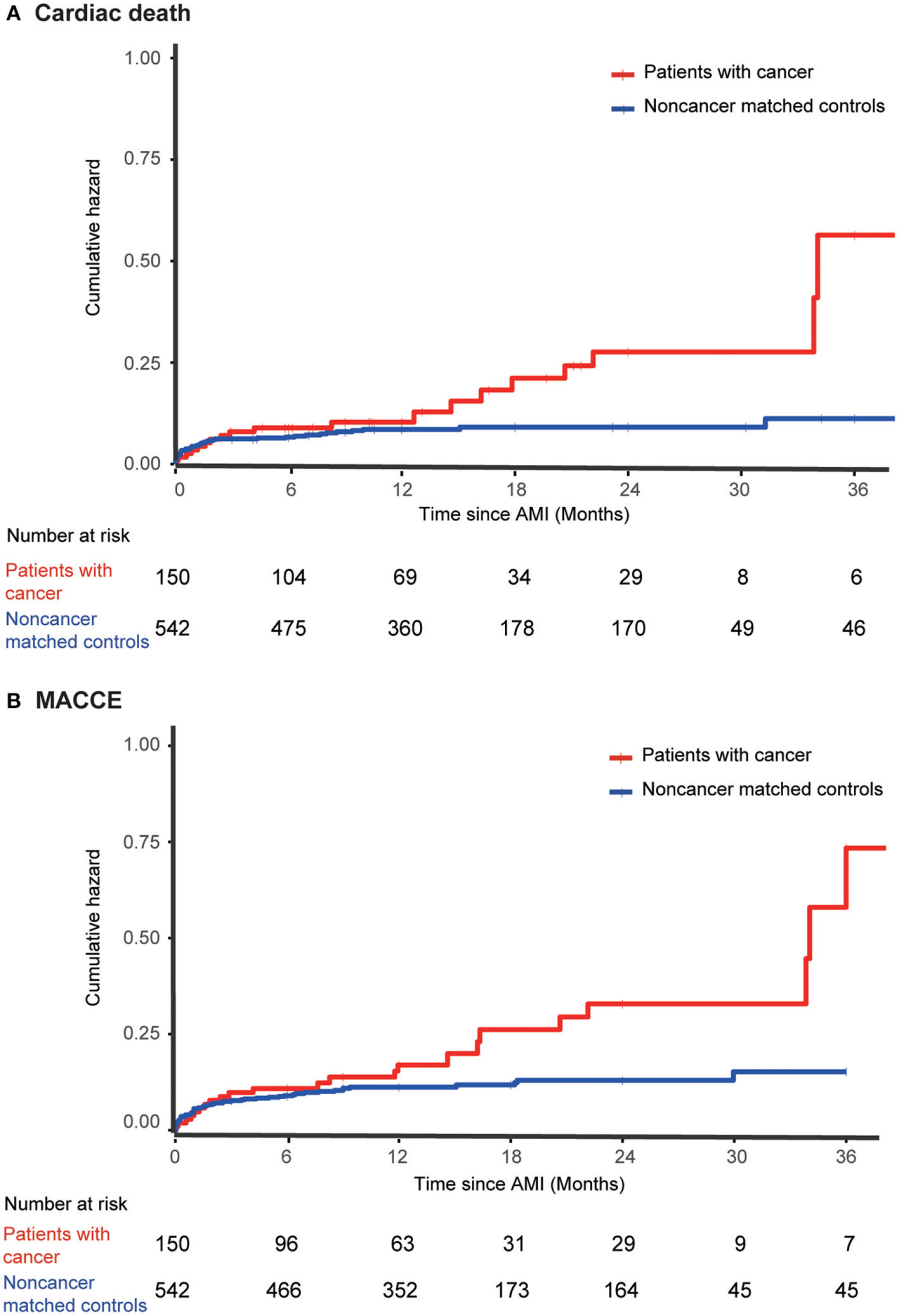
Clinical outcomes among AMI patients with and without cancer. Displayed are the cumulative incidence curves for **(A)** cardiac mortality and **(B)** MACCE for cancer patients vs. controls. AMI, acute myocardial infarction; MACCE, major adverse cardiovascular and cerebrovascular events.

**Figure 2:**
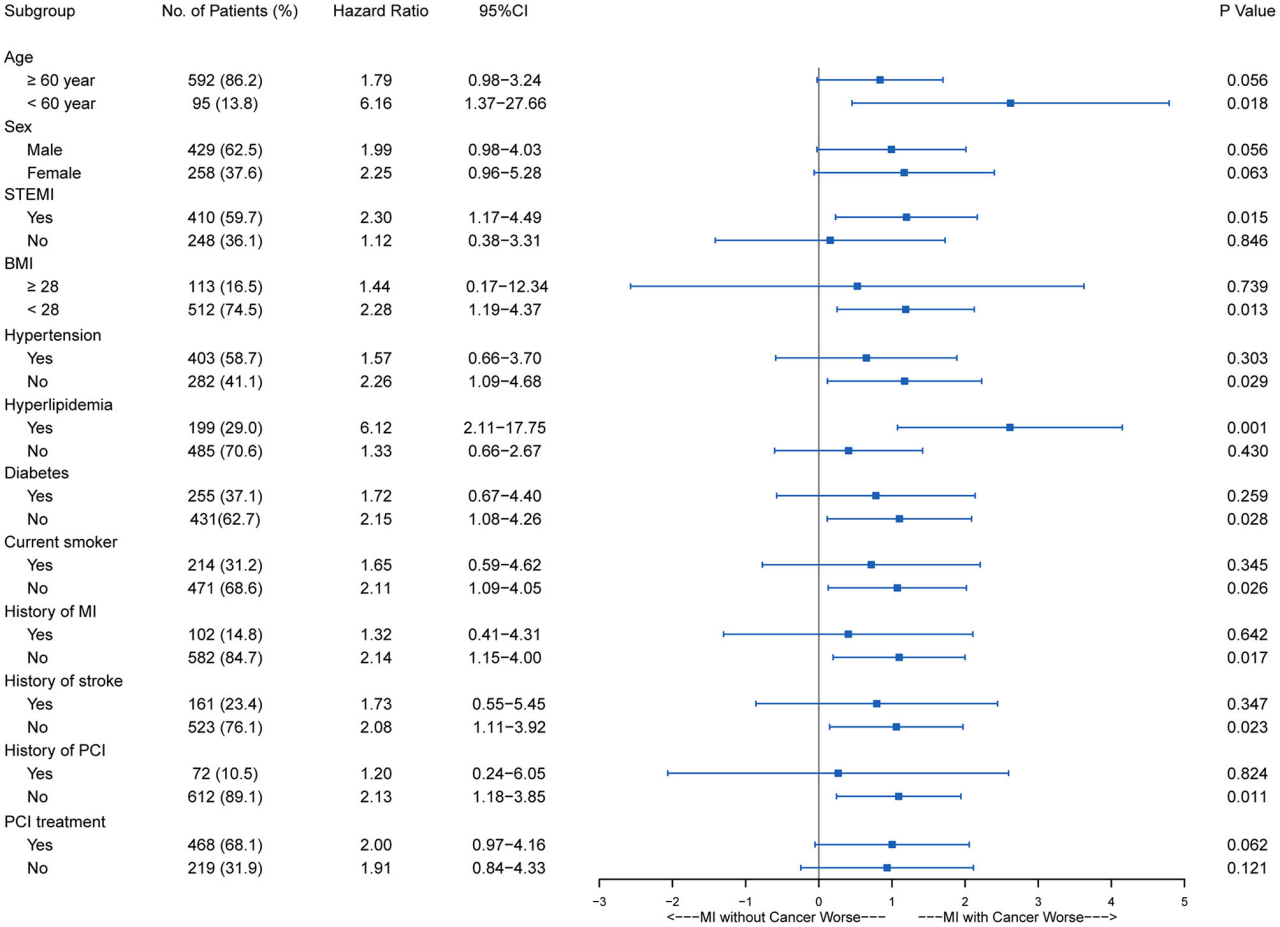
Subgroup stratified analysis of cardiovascular survival among AMI patients with and without cancer. AMI, acute myocardial infarction; BMI, body mass index; CI, confidence interval; MI, myocardial infarction; PCI, percutaneous coronary intervention; STEMI, ST segment elevation myocardial infarction.

With regard to the long-term outcomes, patients with cancer had a significantly higher cumulative incidence of all-cause mortality (22.7 vs. 9.8%; adjusted HR, 2.40; 95% CI, 1.52–3.79; *P* < 0.001) (Supplementary Figure S1) and cardiovascular mortality (14.0 vs. 8.3%; adjusted HR, 1.934; 95% CI, 1.11–3.39; *P* = 0.021) (Figure 1; Supplementary Table S2). MACCE were also significantly higher in the patients with cancer than in the matched non-cancer group (16.0 vs. 10.3%; adjusted HR, 1.98; 95% CI, 1.21–3.26; *P* = 0.007). Moreover, there was no significant difference in MI (2.7 vs. 1.7%; adjusted HR, 1.64; 95% CI, 0.50–5.41; *P* = 0.419), stroke (0.7 vs. 0.9%; adjusted HR, 0.84; 95% CI, 0.10–7.34; *P* = 0.876), and revascularization (1.3 vs. 4.6%; adjusted HR, 0.259; 95% CI, 0.061–1.097; *P* = 0.067) between patients with or without cancer. Cardiovascular mortality tended to be similar across all pre-specified subgroups (Figure 2), as was all-cause mortality and MACCE (Supplementary Figures S2, S3).

Among 150 patients with cancer, 52 had a historical cancer diagnosis beyond 5 years before AMI, 59 had a historical cancer diagnosis within 5 years before AMI, and the other 39 had a current cancer diagnosis after AMI. The incidences of all-cause mortality, cardiovascular mortality and MACCE were significantly higher among patients with a historical cancer diagnosis within 5 years than among those without cancer (adjusted HR, 3.38; 95% CI, 1.88–6.04; *P* < 0.001; adjusted HR, 2.59; 95% CI, 1.25–5.35; *P* = 0.010; and adjusted HR, 2.66; 95% CI, 1.39–5.11; *P* = 0.003, respectively) (Table 2). A similar pattern was observed for all-cause mortality among patients with a current cancer diagnosis (adjusted HR, 2.71; 95% CI, 1.25–5.88; *P* = 0.012).

**Table 2:** Outcomes according to the timing of the cancer diagnosis.

**Outcome**	**Events** **(N/All)**	**HR (95%CI)** **(Cancer vs.** **no cancer)**	***P-*value**	**Adjusted HR^a^ (95%CI)** **(Cancer vs.** **no cancer)**	**Adjusted** ***P-*value^a^**
**All-cause death**					
All cancer	34/87	2.465 (1.581–3.843)	<0.001	2.402 (1.523–3.789)	<0.001
History > 5 years	8	1.460 (0.692–3.081)	0.320	1.453 (0.685–3.082)	0.331
History ≤ 5 years	16	3.271 (1.833–5.836)	<0.001	3.375 (1.884–6.044)	<0.001
Current	10	3.099 (1.523–6.304)	0.002	2.708 (1.248–5.875)	0.012
**Cardiac death**					
All cancer	21/66	2.075 (1.206–3.569)	0.008	1.934 (1.105–3.386)	0.021
History > 5 years	6	1.717 (0.730–4.040)	0.216	1.649 (0.697–3.904)	0.255
History ≤ 5 years	10	2.550 (1.239–5.246)	0.011	2.589 (1.253–5.348)	0.010
Current	5	1.879 (0.674–5.241)	0.228	1.348 (0.406–4.473)	0.626
**MACCE**					
All cancer	24/80	2.302 (1.251–3.303)	0.004	1.982 (1.205–3.261)	0.007
History > 5 years	7	1.530 (0.697–3.358)	0.289	1.542 (0.700–3.397)	0.282
History ≤ 5 years	11	2.571 (1.346–4.910)	0.004	2.661 (1.385–5.111)	0.003
Current	6	2.031 (0.814–5.071)	0.129	1.647 (0.578–4.692)	0.350

*CI, confidence interval; HR, hazard ratio; MACCE, major adverse cardiovascular and cerebrovascular events*.

a *HRs were calculated using adjustments for history of coronary heart disease, history of myocardial infarction, history of percutaneous coronary intervention and history of chronic kidney disease*.

## Discussion

The main findings of this study are as follows: (a) among AMI patients, those with cancer were generally older and more often presented with comorbidities than those without cancer; (b) patients with cancer tended to be treated with conservative medical strategies with a weaker P2Y12 inhibitor in dual anti-platelet therapy (DAPT) and less PCI; (c) patients with cancer had a significantly higher incidence of cardiovascular mortality and MACCE; (d) patients with a historical cancer diagnosis within 5 years had a higher risk of cardiovascular ischemic events.

### Patients With Cancer Tended to Be Treated With Less PCI

We found that patients with cancer are less likely to undergo PCI treatment during hospitalization than those without cancer, and they were also less likely to undergo revascularization during follow-up. According to previous data, patients with active cancer have ~2- and 3-fold higher risks of 90 days for readmission with AMI or major bleeding after PCI, respectively, than patients without cancer (18). Thus, clinicians are often wary of performing invasive therapies in patients with cancer. However, data from large retrospective studies showed that PCI results in significantly lower risks of in-hospital all-cause mortality and MACCE than conservative treatment, irrespective of whether the patient had a cancer diagnosis, and PCI did not increase the risk of in-hospital complications, including massive bleeding and stroke (19). To date, there has been no large randomized trial to assess the benefits and risks of invasive and conservative approaches to treating AMI in patients with cancer, and such patients are often excluded from clinical trials. The current guidelines recommend that percutaneous revascularization should be considered even in cancer patients with an expected survival duration of <1 year (20). Balloon angioplasty without stents are recommended to limit the duration of antiplatelet therapy. If stents need to be used, those with fast reendothelialization rates may be a better choice.

### Clinicians Prefer Conservative Clopidogrel Rather Than Ticagrelor for Aspirin-Based DAPT

The coexistence of high risks of ischemia and major bleeding presents a challenge for clinicians when treating AMI patients with cancer with regard to antiplatelet therapy. When faced with this dilemma, clinicians prefer conservative approaches with regard to aspirin-based DAPT. A less potent P2Y12 inhibitor, namely, clopidogrel rather than ticagrelor, was administered to AMI patients with cancer, but there is a lack of reliable evidence to confirm the greater benefits of clopidogrel among such high-risk patients.

### Patients With Cancer Had a Significantly Higher Incidence of Adverse Cardiovascular Outcomes Than Those Without Cancer

A previous study that included 6,563,255 AMI patients revealed that patients with cancer, irrespective of the cancer type, had higher risks of in-hospital mortality, MACCE, and stroke than those without cancer (21). Inflammation plays a vital role in the progression of both cancer and atherosclerotic lesions (including CHD) (22). Although the mechanism underlying this association is unclear, we propose that local malignancies might increase vascular wall inflammation by releasing inflammatory cytokines and that this circulatory inflammation might subsequently lead to progressive coronary atherosclerosis. In addition, cardiotoxicity can be a major complication of cancer treatment, radiotherapy is recognized as a cardiovascular risk factor among patients with cancer, and many anticancer drugs (anthracyclines, vinca alkaloid anti-metabolites, and biologics) are known to be closely associated with acute early and late cardiovascular adverse events. Perhaps because of the overlap of common risk factors for cancer and CHD and the susceptibility to atherosclerosis caused by oncology treatments (such as radiation therapy or tyrosine kinase inhibitors), patients with cancer tend to exhibit a relatively higher cardiovascular risk. In particular, there was no significant difference in cardiovascular mortality and MACCE for 1 year, but we found that there was no significant difference in cardiovascular mortality and MACCE for 1 year (Supplementary Table S3), and the 3-year incidences of all-cause mortality, cardiovascular mortality and MACCE were significantly higher among patients with cancer than among those without cancer (Supplementary Table S2). These problems highlight the fact that cardiovascular diseases become more important during the long-term survival of patients with cancer. Advances in screening, big data, targeted and immune therapies, and significant new knowledge of cancer biology are changing the prevention, detection, diagnosis, treatment and survival of cancer. However, the current treatments are still mostly based on extrapolation from non-cancer patient data, and there remain some gaps in achieving the goal of personalized treatment for AMI patients with cancer.

### Patients With a Historical Cancer Diagnosis Within 5 Years Had a Higher Risk of Adverse Cardiovascular Outcomes Among All Subgroups

Furthermore, subgroup analysis was performed according to the time between the diagnosis of cancer and the occurrence of AMI. The results showed that the incidences of all-cause death, cardiovascular death and MACCE in the group with a historical cancer diagnosis within 5 years were significantly higher than in those without cancer, and the risks in that subgroup were the highest among all subgroups. This connection is not accidental, and a large-scale study from Sweden also found that patients with cancer had the highest risk of CHD in the first 6 months after diagnosis (23). Another previous study reported similar results: the risks of in-hospital mortality and MACCEs were higher by at least 50% among AMI patients with a current cancer diagnosis than among those without cancer, whereas they were not higher among patients with a historical cancer diagnosis (21). Our findings also underscore the importance of vigilance in cardiovascular risk monitoring after cancer treatment. It is critical to continue assessing the risk of potential cardiovascular events among patients with cancer, and future randomized trials are needed to evaluate the effectiveness of such surveillance.

### Limitations

(a) We acknowledge all limitations inherent to a retrospective, single-center study, which restrict the generalization of our findings and the inference of causality. (b) The overall cancer population was relatively small, and the subgroups related to cardiovascular safety concerns were potentially underpowered. In addition, the patients with cancer were a heterogeneous population with different cancer types and stages, and the sample size was too small to evaluate each cancer type separately. (c) Although the data for AMI patients were abundant, the lack of complete cancer history and cancer types may be considered a limitation of this study. The missing data on cancer metastasis, stages, and cancer treatment limits the further understanding of the differences in outcomes between AMI patients with cancer and those without cancer.

## Conclusions

AMI patients with cancer tended to have a significantly higher risk of cardiovascular adverse outcomes than those without cancer. Given the limited evidence-based guidance, clinicians are more likely to empirically initiate conservative treatment when faced with the dilemma of ischemia and the risk of major bleeding. Thus, it is vital to raise awareness of cardiovascular risk management and continuously optimize cardiovascular treatment among patients with cancer.

## Data Availability Statement

The raw data supporting the conclusions of this article will be made available by the authors, without undue reservation.

## Ethics Statement

The studies involving human participants were reviewed and approved by the Ethics Committee of Harbin Medical University. The patients/participants provided their written informed consent to participate in this study.

## Author Contributions

XP, ZW, BY, and JT: study concept and design. XP, ZW, MC, YZ, YT, LY, and WN: acquisition of data. XP and ZW: analysis and interpretation of data and drafting of the manuscript. MC, SW, ZQ, and SZ: critical revision of the manuscript for intellectual content. XP and ZW: statistical analysis. BY and JT: obtaining funding. All authors gave final approval and agreed to be accountable for all aspects of the work, ensuring integrity and accuracy.

## Funding

This work was supported by grants from the National Natural Science Foundation of China (Grant Nos. 91739113, 81971715 to JT and 81827806 to BY), the Applied Technology Research and Development Program of Heilongjiang Province (Grant No. GA20C007 to JT), the National Key R&D Program of China (Grant No. 2016YFC1301100 to BY), the Fok Ying-Tong Education Foundation for Young Teachers (171032 to JT) and the Foundation of Guangxi Key Laboratory of Diabetic Systems Medicine (GKLCDSM-20200101-01 to JT).

## Conflict of Interest

The authors declare that the research was conducted in the absence of any commercial or financial relationships that could be construed as a potential conflict of interest.

## Publisher's Note

All claims expressed in this article are solely those of the authors and do not necessarily represent those of their affiliated organizations, or those of the publisher, the editors and the reviewers. Any product that may be evaluated in this article, or claim that may be made by its manufacturer, is not guaranteed or endorsed by the publisher.

## Supplementary Material

Supplementary Figure S1 Clinical outcomes among AMI patients with and without cancer. Displayed are the cumulative incidence curves for **(A)** all-cause mortality, **(B)** MI, **(C)** stroke, and **(D)** revascularization for cancer patients vs. controls. AMI, acute myocardial infarction; MI, myocardial infarction.

Supplementary Figure S2 Subgroup stratified analysis of all-cause mortality among AMI patients with and without cancer. AMI, acute myocardial infarction; BMI, body mass index; CI, confidence interval; MI, myocardial infarction; PCI, percutaneous coronary intervention; STEMI, ST segment elevation myocardial infarction.

Supplementary Figure S3 Subgroup stratified analysis of MACCE among AMI patients with and without cancer. AMI, acute myocardial infarction; BMI, body mass index; CI, confidence interval; MACCE, major adverse cardiovascular and cerebrovascular events; MI, myocardial infarction; PCI, percutaneous coronary intervention; STEMI, ST segment elevation myocardial infarction.

Supplementary Table S1 Pathological types in the 150 patients with cancer.

Supplementary Table S2 Cumulative incidence of outcomes among AMI patients with and without cancer.

Supplementary Table S3 Cumulative incidence of 1-year outcomes among AMI patients with and without cancer.
