# Supplementary figures and images for: A Concomitant Cancer Diagnosis Is Associated With Poor Cardiovascular Outcomes Among Acute Myocardial Infarction Patients
Source: Front Cardiovasc Med. 2022 Feb 17;9:758324. doi: 10.3389/fcvm.2022.758324 (PMC8891500; doi:10.3389/fcvm.2022.758324)

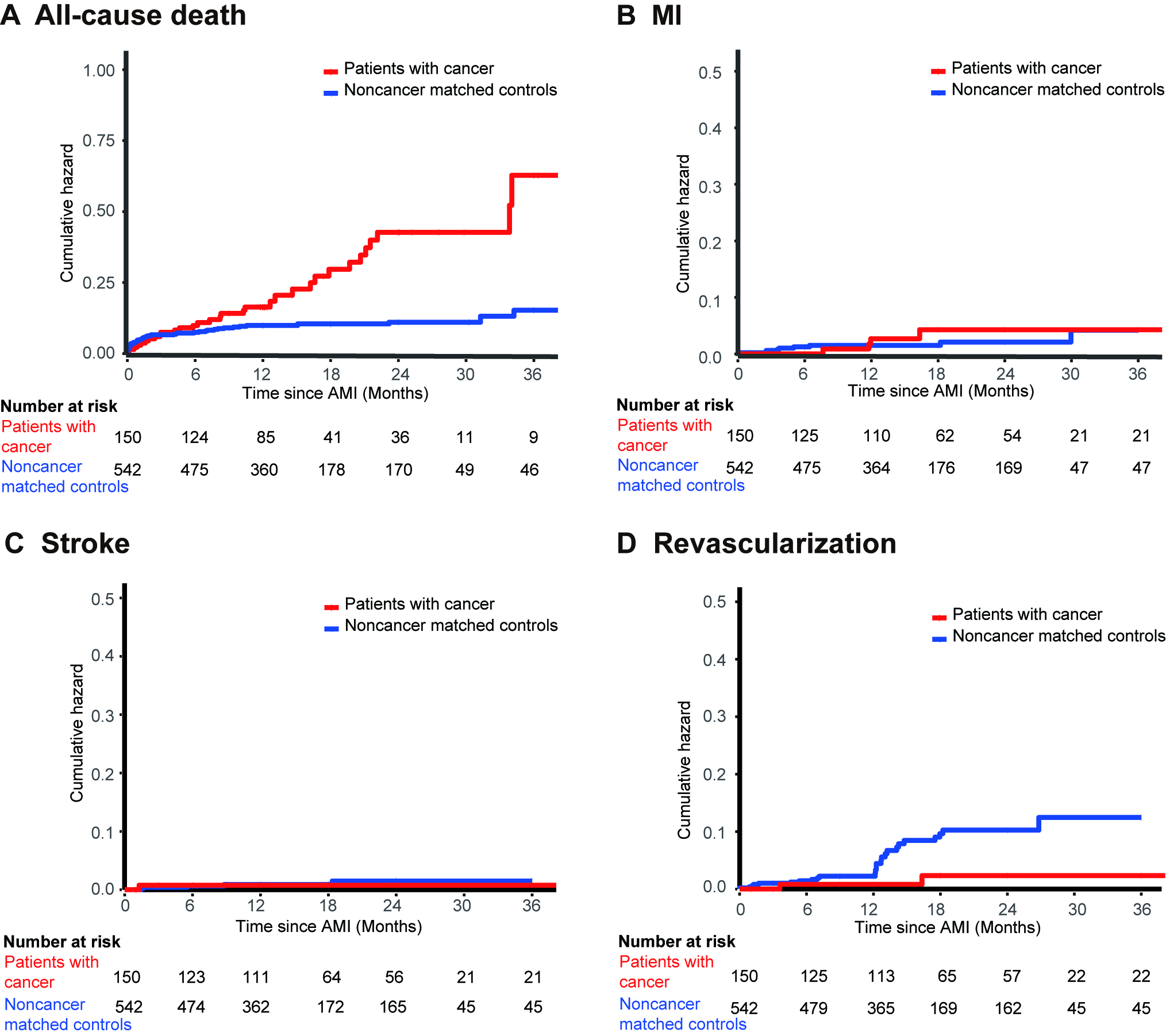

Supplement: Supplementary Figure S1 — Clinical outcomes among AMI patients with and without cancer. Displayed are the cumulative incidence curves for (A) all-cause mortality, (B) MI, (C) stroke, and (D) revascularization for cancer patients vs. controls. AMI, acute myocardial infarction; MI, myocardial infarction. [file Image_1.TIF]

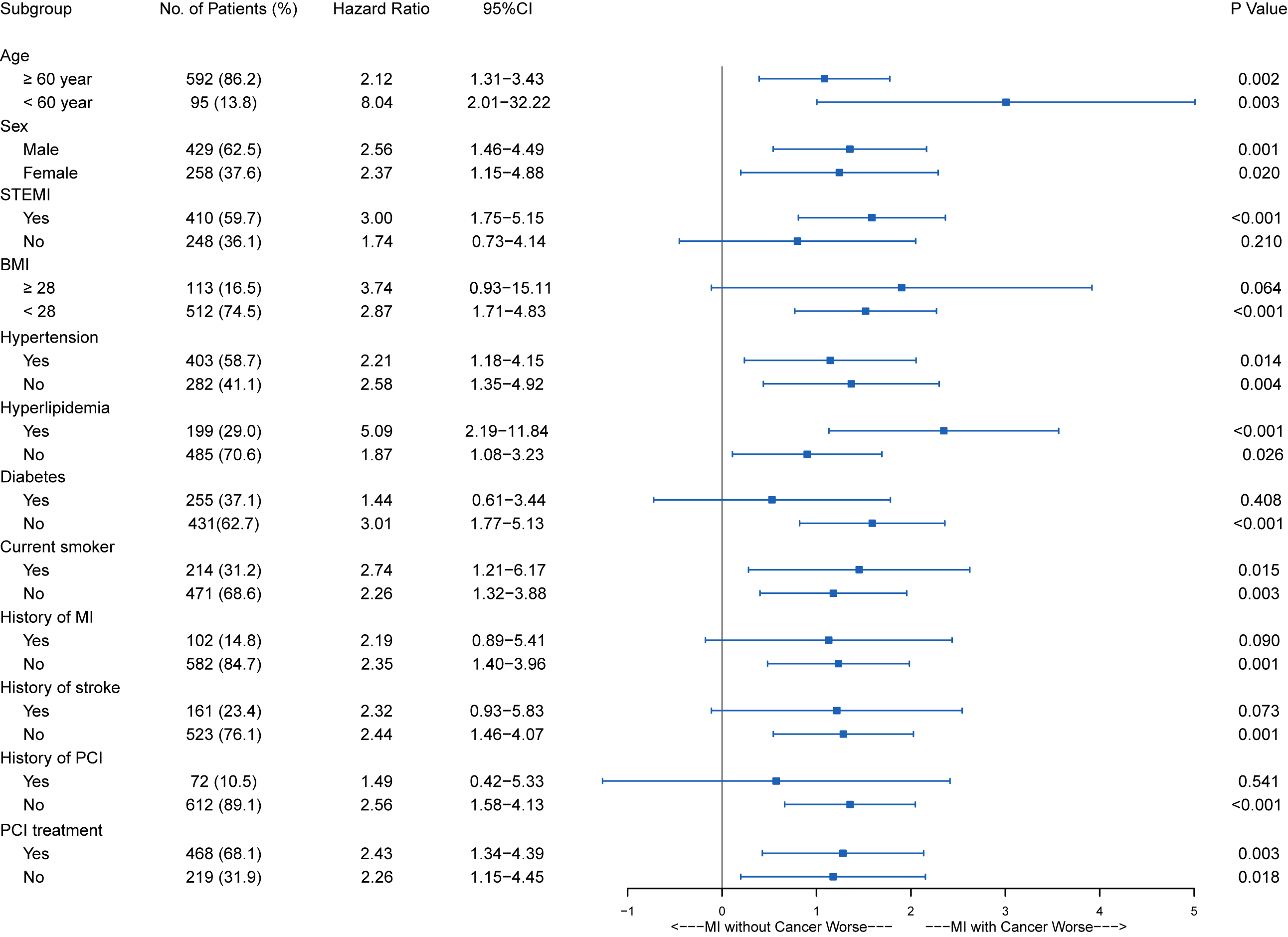

Supplement: Supplementary Figure S2 — Subgroup stratified analysis of all-cause mortality among AMI patients with and without cancer. AMI, acute myocardial infarction; BMI, body mass index; CI, confidence interval; MI, myocardial infarction; PCI, percutaneous coronary intervention; STEMI, ST segment elevation myocardial infarction. [file Image_2.TIF]

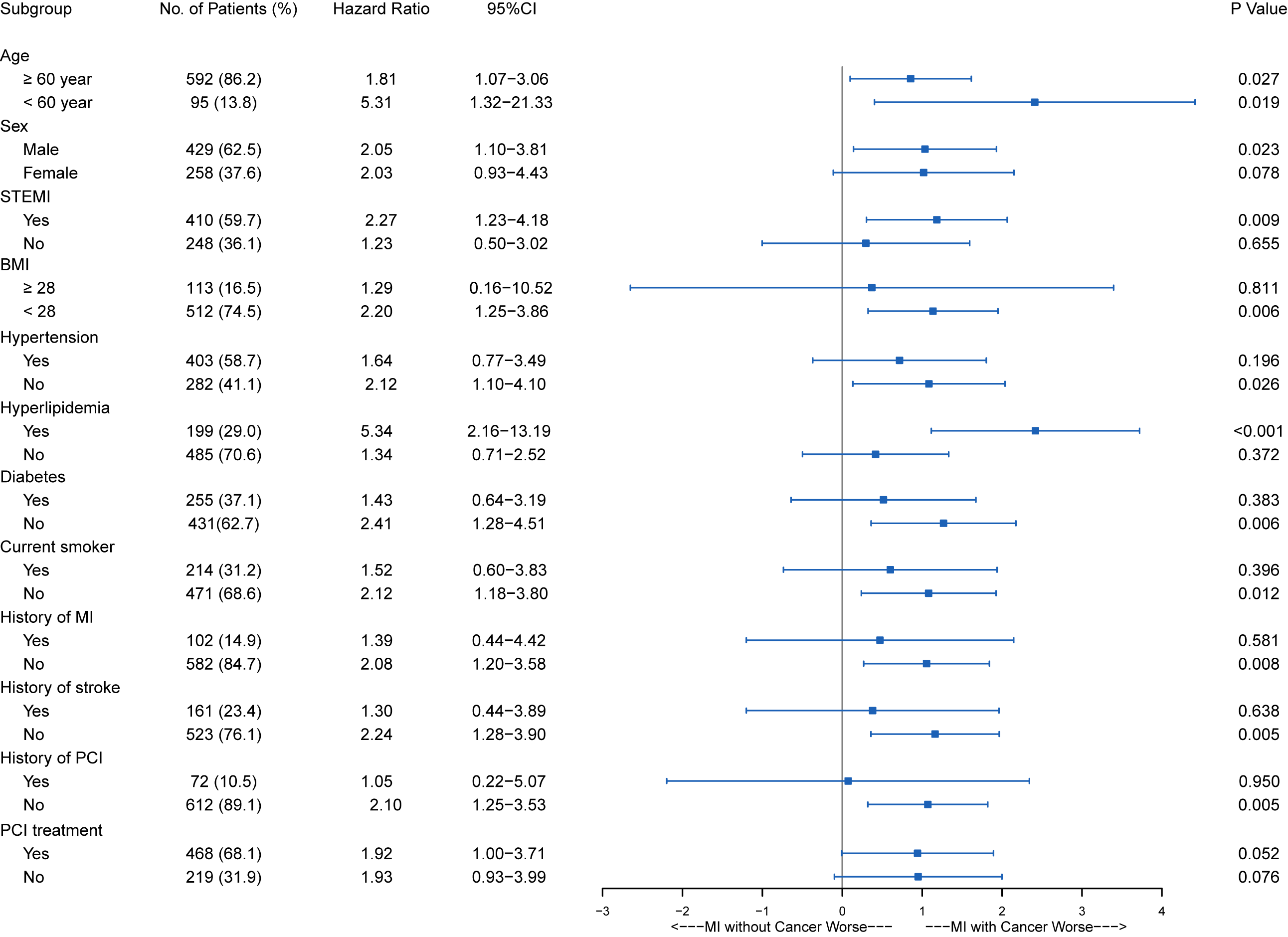

Supplement: Supplementary Figure S3 — Subgroup stratified analysis of MACCE among AMI patients with and without cancer. AMI, acute myocardial infarction; BMI, body mass index; CI, confidence interval; MACCE, major adverse cardiovascular and cerebrovascular events; MI, myocardial infarction; PCI, percutaneous coronary intervention; STEMI, ST segment elevation myocardial infarction. [file Image_3.TIF]
