# Supplementary material for: A Concomitant Cancer Diagnosis Is Associated With Poor Cardiovascular Outcomes Among Acute Myocardial Infarction Patients
Source: Front Cardiovasc Med. 2022 Feb 17;9:758324. doi: 10.3389/fcvm.2022.758324 (PMC8891500; doi:10.3389/fcvm.2022.758324)
Supplement: Supplementary Table S1 — Pathological types in the 150 patients with cancer. [file Table_1.DOCX]

**Table S1. Pathological types in the 150 patients with cancer.**

| **Type of tumor** | **Patients, n (%)** |
| --- | --- |
| Lung | 31(20.7) |
| Colorectum | 21(14.0) |
| Stomach | 19(12.7) |
| Breast | 15(10.0) |
| Urinary bladder | 13(8.7) |
| Kidney | 8(5.3) |
| Thyroid | 7(4.7) |
| Liver | 6(4.0) |
| Cervix uteri | 4(2.7) |
| Lymph | 4(2.7) |
| Bone | 2(1.3) |
| Cholangiocarcinoma | 2(1.3) |
| Esophagus | 2(1.3) |
| Larynx | 2(1.3) |
| Pancreas | 2(1.3) |
| Skin | 2(1.3) |
| Eye | 1(0.7) |
| Endometrium | 1(0.7) |
| Gingiva | 1(0.7) |
| Multiple Myeloma | 1(0.7) |
| Nasopharynx | 1(0.7) |
| Oral cavity | 1(0.7) |
| Ovary | 1(0.7) |
| Prostate | 1(0.7) |
| Paranephros | 1(0.7) |
| Testis | 1(0.7) |
