# Supplementary material for: A Concomitant Cancer Diagnosis Is Associated With Poor Cardiovascular Outcomes Among Acute Myocardial Infarction Patients
Source: Front Cardiovasc Med. 2022 Feb 17;9:758324. doi: 10.3389/fcvm.2022.758324 (PMC8891500; doi:10.3389/fcvm.2022.758324)
Supplement: Supplementary Table S2 — Cumulative incidence of outcomes among AMI patients with and without cancer. [file Table_2.DOCX]

**Table S2.** **Cumulative incidence of outcomes among AMI patients with and without cancer**

| **Outcome** | **No cancer**  **(N = 542)** | **Cancer**  **(N = 150)** | **HR (95% CI)**  **(Cancer vs.** **No cancer)** | ***P v*alue** | **Adjusted HR ^a^ (95% CI)**  **(Cancer vs.** **No cancer)** | **Adjusted *P v*alue ^a^** |
| --- | --- | --- | --- | --- | --- | --- |
| All-cause death | 53 (9.8) | 34 (22.7) | 2.465 (1.581-3.843) | <0.001 | 2.402 (1.523-3.789) | <0.001 |
| Cardiac death | 45 (8.3) | 21 (14.0) | 2.075 (1.206-3.569) | 0.008 | 1.934 (1.105-3.386) | 0.021 |
| MACCE | 56 (10.3) | 24 (16.0) | 2.302 (1.251-3.303) | 0.004 | 1.982 (1.205-3.261) | 0.007 |
| MI | 9 (1.7) | 4 (2.7) | 1.483 (0.456-4.831) | 0.513 | 1.638 (0.496-5.411) | 0.419 |
| Stroke | 5 (0.9) | 1 (0.7) | 0.710 (0.083-6.084) | 0.755 | 0.841 (0.096-7.336) | 0.876 |
| Revascularization | 25 (4.6) | 2 (1.3) | 0.238 (0.056-1.005) | 0.051 | 0.259 (0.061-1.097) | 0.067 |

Values are n (%)

Abbreviations: AMI, acute myocardial infarction; CI, confidence interval; HR, hazard ratio; MACCE, major adverse cardiovascular and cerebrovascular events; MI, myocardial infarction.

^a^ HRs were calculated using adjustments for history of coronary heart disease, history of MI, history of percutaneous coronary intervention and history of chronic kidney disease.
