# Supplementary material for: A Concomitant Cancer Diagnosis Is Associated With Poor Cardiovascular Outcomes Among Acute Myocardial Infarction Patients
Source: Front Cardiovasc Med. 2022 Feb 17;9:758324. doi: 10.3389/fcvm.2022.758324 (PMC8891500; doi:10.3389/fcvm.2022.758324)
Supplement: Supplementary Table S3 — Cumulative incidence of 1-year outcomes among AMI patients with and without cancer. [file Table_3.DOCX]

**Table S2.** **Cumulative incidence of 1-year outcomes among AMI patients with and without cancer**

| **Outcome** | **No cancer**  **(N = 542)** | **Cancer**  **(N = 150)** | **HR (95% CI)**  **(Cancer vs.** **No cancer)** | ***P v*alue** | **Adjusted HR ^a^ (95% CI)**  **(Cancer vs.** **No cancer)** | **Adjusted *P v*alue ^a^** |
| --- | --- | --- | --- | --- | --- | --- |
| All-cause death | 39 (7.2) | 19 (12.7) | 1.765 (1.020-3.055) | 0.042 | 1.733 (0.946-3.177) | 0.075 |
| Cardiac death | 32 (5.9) | 10 (6.7) | 1.121 (0.551-2.281) | 0.752 | 0.885 (0.380-2.062) | 0.777 |
| MACCE | 44 (8.1) | 14 (9.3) | 1.146 (0.628-2.091) | 0.657 | 1.068 (1.205-3.261) | 0.850 |
| MI | 6 (1.1) | 3 (2.0) | 1.862 (0.466-7.448) | 0.379 | 2.570 (0.662-10.621) | 0.192 |
| Stroke | 4 (0.7) | 1 (0.7) | 0.912 (0.102-8.163) | 0.934 | 1.110 (0.124-9.962) | 0.926 |
| Revascularization | 14 (2.6) | 1 (0.7) | 0.257 (0.034-1.955) | 0.189 | 0.336 (0.044-2.565) | 0.293 |

Values are n (%)

Abbreviations: AMI, acute myocardial infarction; CI, confidence interval; HR, hazard ratio; MACCE, major adverse cardiovascular and cerebrovascular events; MI, myocardial infarction.

^a^ HRs were calculated using adjustments for history of coronary heart disease, history of MI, history of percutaneous coronary intervention and history of chronic kidney disease.
